# 5-(1*H*-1,2,3-Benzotriazol-1-ylmeth­yl)-3-phenyl-1,2,4-oxadiazole

**DOI:** 10.1107/S1600536808019879

**Published:** 2008-07-05

**Authors:** Shu-Qing Xu, Jia-Ming Li

**Affiliations:** a Laboratory of Beibu Gulf Marine Protection and Exploitation, Department of Chemistry and Biology, Qinzhou University, Qinzhou, Guangxi 535000, People’s Republic of China

## Abstract

In the title mol­ecule, C_15_H_11_N_5_O, the 1,2,3-benzotriazole and 3-phenyl-1,2,4-oxadiazole units are individually essentially planar and the dihedral angle between them is 80.2 (2)°. In the crystal structure, mol­ecules are connected *via* weak inter­molecular C—H⋯N hydrogen bonds to form two-dimensional sheets.

## Related literature

For related literature, see: Batista *et al.* (2000 ▶); Wardell *et al.* (2003 ▶); Srinivasan *et al.* (2007 ▶); Wang *et al.* (2004*a* ▶,*b* ▶,*c* ▶, 2007 ▶); Romero (2001 ▶); Terashita *et al.* (2002 ▶); Zen *et al.* (1983 ▶).
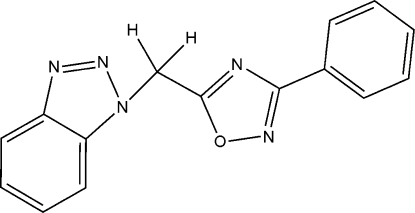


## Experimental

### 

#### Crystal data

C_15_H_11_N_5_O
*M* *_r_* = 277.29
Monoclinic, 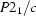

*a* = 4.7009 (13) Å
*b* = 11.100 (3) Å
*c* = 25.265 (7) Å
β = 95.234 (6)°
*V* = 1312.8 (6) Å^3^
*Z* = 4
Mo *K*α radiation
μ = 0.09 mm^−1^
*T* = 295 K
0.18 × 0.14 × 0.12 mm

#### Data collection

Bruker SMART diffractometer
Absorption correction: multi-scan (*SADABS*; Sheldrick, 1996 ▶) *T*_min_ = 0.983, *T*_max_ = 0.989
6803 measured reflections
2322 independent reflections
1324 reflections with *I* > 2σ(*I*)
*R*_int_ = 0.053

#### Refinement

*R*[*F* ^2^ > 2σ(*F* ^2^)] = 0.050
*wR*(*F* ^2^) = 0.103
*S* = 1.02
2322 reflections
190 parameters
H-atom parameters constrained
Δρ_max_ = 0.13 e Å^−3^
Δρ_min_ = −0.19 e Å^−3^

### 

Data collection: *SMART* (Bruker, 2003 ▶); cell refinement: *SAINT* (Bruker, 2003 ▶); data reduction: *SAINT*; program(s) used to solve structure: *SHELXS97* (Sheldrick, 2008 ▶); program(s) used to refine structure: *SHELXL97* (Sheldrick, 2008 ▶); molecular graphics: *SHELXTL* (Sheldrick, 2008 ▶); software used to prepare material for publication: *SHELXTL*.

## Supplementary Material

Crystal structure: contains datablocks global, I. DOI: 10.1107/S1600536808019879/lh2650sup1.cif

Structure factors: contains datablocks I. DOI: 10.1107/S1600536808019879/lh2650Isup2.hkl

Additional supplementary materials:  crystallographic information; 3D view; checkCIF report

## Figures and Tables

**Table 1:** Hydrogen-bond geometry (Å, °)

*D*—H⋯*A*	*D*—H	H⋯*A*	*D*⋯*A*	*D*—H⋯*A*
C9—H9*B*⋯N5^i^	0.97	2.59	3.443 (3)	147
C9—H9*A*⋯N2^ii^	0.97	2.60	3.466 (3)	149

Symmetry codes: (i) 
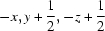
; (ii) 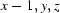.

**Fig. 1.**
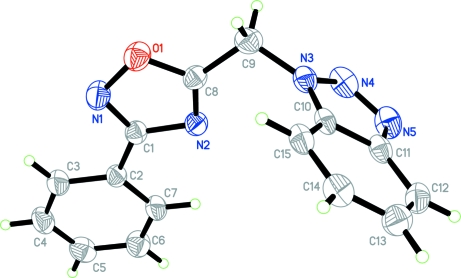
The molecular structure with displacement ellipsoids at the 30% probability level.

**Fig. 2.**
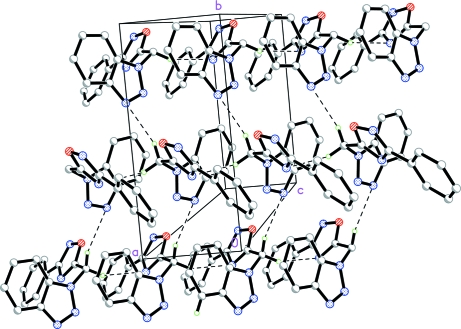
Part of the crystal structure showing hydrogen bonds as dashed lines. H atoms, except for those involved in hydrogen bonds, are not included.

## supplementary crystallographic information

### Comment

Synthesis of 1,2,4-oxadiazole derivatives has attracted a great interest due to their pharmacological properties such as intrinsic analgesic (Zen *et al.*, 1983; Terashita *et al.*, 2002) and antipicornaviral (Romero, 2001) effects. Wang *et al.* (2004a,b,c;2007) have described the synthesis and crystal structures of a series of these types of compounds. Herein, we report the synthesis and crystal structure of the title compound, (I), containing both 1,2,4-oxadiazole and 1,2,3-benzotriazole organic functional groups. The molecular structure of (I) is shown in Fig. 1. The title molecule can be considered as two rings systems: the 3-phenyl-1,2,4-oxadiazol-5-yl (A) and  benzotriazole (B). All atoms in A, B are individually essentially planar with dihedral angles of 4.4 (2) ° and 0.5 (2) ° between the rings in each, respectively. The dihedral angle between A and B is 80.2 (2) °. This conformation presents no nonbonded interactions (Batista *et al.*,2000). Molecules are connected *via* weak intermolecular C—H···N hydrogen bonds (Table. 1 and Fig. 2) to form two-dimensional sheets.

### Experimental

Reagents and solvents used were of commercially available quality. 1,2,3-Benzotriazole (1 mmol) was dissolved in acetonitrile (80 ml) and potassium carbonate (15 mmol) was added followed by 3-phenyl-5-chloromethyl-1,2,4-oxadiazole (1 mmol). The resulting mixture was refluxed for 10 h. After cooling and filtering, the crude title compound was obtained and purified by recrystallization from ethyl acetate. Crystals suitable for X-ray diffraction were obtained by slow evaporation of an ethanol solution of (I).

### Refinement

H atoms were positioned geometrically and refined using a riding model, with C—H = 0.93–0.97 Å and with *U*_iso_(H) = 1.2 times *U*_eq_(C).

### Crystal data

**Table:**

C_15_H_11_N_5_O	*F*_000_ = 576
*M**_r_* = 277.29	*D*_x_ = 1.403 Mg m^−^^3^
Monoclinic, *P*2_1_/*c*	Mo *K*α radiation λ = 0.71073 Å
Hall symbol: -P 2ybc	Cell parameters from 760 reflections
*a* = 4.7009 (13) Å	θ = 2.5–19.5º
*b* = 11.100 (3) Å	µ = 0.09 mm^−^^1^
*c* = 25.265 (7) Å	*T* = 295 K
β = 95.234 (6)º	Block, colorless
*V* = 1312.8 (6) Å^3^	0.18 × 0.14 × 0.12 mm
*Z* = 4	

### Data collection

**Table:**

Bruker SMART diffractometer	2322 independent reflections
Radiation source: fine-focus sealed tube	1324 reflections with *I* > 2σ(*I*)
Monochromator: graphite	*R*_int_ = 0.054
*T* = 295 K	θ_max_ = 25.0º
φ and ω scans	θ_min_ = 1.6º
Absorption correction: multi-scan(SADABS; Sheldrick, 1996)	*h* = −5→5
*T*_min_ = 0.983, *T*_max_ = 0.989	*k* = −13→13
6803 measured reflections	*l* = −20→29

### Refinement

**Table:**

Refinement on *F*^2^	Secondary atom site location: difference Fourier map
Least-squares matrix: full	Hydrogen site location: inferred from neighbouring sites
*R*[*F*^2^ > 2σ(*F*^2^)] = 0.050	H-atom parameters constrained
*wR*(*F*^2^) = 0.103	*w* = 1/[σ^2^(*F*_o_^2^) + (0.036*P*)^2^ + 0.0112*P*] where *P* = (*F*_o_^2^ + 2*F*_c_^2^)/3
*S* = 1.02	(Δ/σ)_max_ < 0.001
2322 reflections	Δρ_max_ = 0.13 e Å^−^^3^
190 parameters	Δρ_min_ = −0.19 e Å^−^^3^
Primary atom site location: structure-invariant direct methods	Extinction correction: none

### Special details

**Table:**

Geometry. All e.s.d.'s (except the e.s.d. in the dihedral angle between two l.s. planes) are estimated using the full covariance matrix. The cell e.s.d.'s are taken into account individually in the estimation of e.s.d.'s in distances, angles and torsion angles; correlations between e.s.d.'s in cell parameters are only used when they are defined by crystal symmetry. An approximate (isotropic) treatment of cell e.s.d.'s is used for estimating e.s.d.'s involving l.s. planes.
Refinement. Refinement of *F*^2^ against ALL reflections. The weighted *R*-factor *wR* and goodness of fit *S* are based on *F*^2^, conventional *R*-factors *R* are based on *F*, with *F* set to zero for negative *F*^2^. The threshold expression of *F*^2^ > σ(*F*^2^) is used only for calculating *R*-factors(gt) *etc*. and is not relevant to the choice of reflections for refinement. *R*-factors based on *F*^2^ are statistically about twice as large as those based on *F*, and *R*- factors based on ALL data will be even larger.

### Fractional atomic coordinates and isotropic or equivalent isotropic displacement parameters (Å^2^)

**Table:**

	*x*	*y*	*z*	*U*_iso_*/*U*_eq_	
O1	0.1731 (4)	0.96111 (14)	0.37087 (7)	0.0620 (5)	
N1	0.3748 (5)	0.94501 (18)	0.41561 (8)	0.0626 (6)	
N2	0.4253 (4)	0.80085 (17)	0.35428 (7)	0.0477 (5)	
N3	0.1502 (4)	0.80219 (18)	0.24592 (8)	0.0488 (5)	
N4	0.1194 (5)	0.68098 (19)	0.23986 (9)	0.0626 (6)	
N5	0.2711 (5)	0.64667 (18)	0.20147 (9)	0.0644 (6)	
C1	0.5148 (5)	0.8490 (2)	0.40357 (9)	0.0443 (6)	
C2	0.7410 (5)	0.7967 (2)	0.43988 (9)	0.0445 (6)	
C3	0.8252 (5)	0.8510 (2)	0.48824 (10)	0.0587 (7)	
H3	0.7393	0.9224	0.4976	0.070*	
C4	1.0355 (6)	0.7994 (3)	0.52249 (10)	0.0669 (8)	
H4	1.0927	0.8369	0.5546	0.080*	
C5	1.1613 (6)	0.6933 (3)	0.50957 (11)	0.0675 (8)	
H5	1.3019	0.6586	0.5330	0.081*	
C6	1.0795 (6)	0.6382 (2)	0.46194 (11)	0.0662 (8)	
H6	1.1652	0.5663	0.4532	0.079*	
C7	0.8703 (5)	0.6893 (2)	0.42703 (10)	0.0550 (7)	
H7	0.8157	0.6517	0.3948	0.066*	
C8	0.2200 (5)	0.8714 (2)	0.33742 (9)	0.0460 (6)	
C9	0.0215 (5)	0.8644 (2)	0.28801 (9)	0.0595 (7)	
H9A	−0.1512	0.8225	0.2956	0.071*	
H9B	−0.0313	0.9452	0.2763	0.071*	
C10	0.3265 (5)	0.8462 (2)	0.21070 (9)	0.0428 (6)	
C11	0.4018 (5)	0.7457 (2)	0.18258 (9)	0.0472 (6)	
C12	0.5861 (5)	0.7564 (2)	0.14226 (10)	0.0578 (7)	
H12	0.6374	0.6898	0.1229	0.069*	
C13	0.6871 (5)	0.8689 (3)	0.13269 (10)	0.0630 (7)	
H13	0.8125	0.8788	0.1066	0.076*	
C14	0.6070 (5)	0.9691 (2)	0.16101 (10)	0.0594 (7)	
H14	0.6790	1.0442	0.1529	0.071*	
C15	0.4258 (5)	0.9607 (2)	0.20035 (10)	0.0515 (6)	
H15	0.3724	1.0279	0.2190	0.062*	

### Atomic displacement parameters (Å^2^)

**Table:**

	*U*^11^	*U*^22^	*U*^33^	*U*^12^	*U*^13^	*U*^23^
O1	0.0714 (12)	0.0570 (11)	0.0567 (12)	0.0150 (9)	0.0015 (10)	−0.0011 (10)
N1	0.0734 (15)	0.0596 (14)	0.0533 (15)	0.0106 (12)	−0.0026 (12)	−0.0082 (12)
N2	0.0492 (12)	0.0528 (12)	0.0405 (13)	0.0061 (10)	0.0007 (10)	0.0001 (11)
N3	0.0531 (12)	0.0484 (13)	0.0431 (13)	0.0011 (10)	−0.0055 (10)	0.0021 (11)
N4	0.0712 (15)	0.0494 (15)	0.0642 (16)	−0.0082 (11)	−0.0104 (13)	0.0118 (13)
N5	0.0798 (17)	0.0458 (14)	0.0649 (16)	−0.0017 (12)	−0.0070 (13)	−0.0007 (13)
C1	0.0483 (14)	0.0460 (15)	0.0392 (16)	−0.0039 (12)	0.0066 (12)	0.0000 (13)
C2	0.0461 (14)	0.0511 (15)	0.0370 (14)	−0.0060 (12)	0.0069 (12)	−0.0030 (13)
C3	0.0609 (17)	0.0646 (17)	0.0502 (17)	−0.0045 (14)	0.0033 (14)	−0.0087 (15)
C4	0.0683 (19)	0.086 (2)	0.0451 (17)	−0.0147 (17)	−0.0028 (15)	−0.0076 (17)
C5	0.0631 (18)	0.084 (2)	0.0536 (19)	−0.0015 (17)	−0.0062 (15)	0.0128 (18)
C6	0.0714 (19)	0.0660 (18)	0.0596 (19)	0.0080 (15)	−0.0031 (15)	−0.0014 (16)
C7	0.0584 (16)	0.0633 (17)	0.0421 (15)	−0.0026 (14)	−0.0024 (13)	−0.0053 (14)
C8	0.0496 (15)	0.0492 (16)	0.0401 (16)	0.0014 (13)	0.0087 (13)	0.0022 (13)
C9	0.0555 (16)	0.0702 (18)	0.0519 (17)	0.0105 (13)	0.0005 (14)	0.0054 (15)
C10	0.0470 (14)	0.0412 (14)	0.0375 (14)	−0.0012 (12)	−0.0113 (12)	−0.0017 (13)
C11	0.0563 (15)	0.0371 (14)	0.0449 (15)	0.0025 (13)	−0.0138 (13)	0.0014 (13)
C12	0.0666 (17)	0.0570 (17)	0.0479 (16)	0.0153 (14)	−0.0052 (14)	−0.0071 (14)
C13	0.0647 (18)	0.073 (2)	0.0509 (18)	0.0072 (15)	0.0034 (14)	0.0064 (16)
C14	0.0647 (17)	0.0539 (17)	0.0584 (18)	−0.0072 (14)	−0.0015 (15)	0.0066 (15)
C15	0.0580 (16)	0.0431 (15)	0.0508 (17)	−0.0012 (13)	−0.0095 (13)	−0.0049 (13)

### Geometric parameters (Å, °)

**Table:**

O1—C8	1.338 (3)	C5—H5	0.9300
O1—N1	1.419 (2)	C6—C7	1.382 (3)
N1—C1	1.303 (3)	C6—H6	0.9300
N2—C8	1.284 (3)	C7—H7	0.9300
N2—C1	1.385 (3)	C8—C9	1.491 (3)
N3—N4	1.360 (2)	C9—H9A	0.9700
N3—C10	1.361 (3)	C9—H9B	0.9700
N3—C9	1.446 (3)	C10—C11	1.386 (3)
N4—N5	1.312 (3)	C10—C15	1.387 (3)
N5—C11	1.366 (3)	C11—C12	1.401 (3)
C1—C2	1.460 (3)	C12—C13	1.365 (3)
C2—C3	1.387 (3)	C12—H12	0.9300
C2—C7	1.390 (3)	C13—C14	1.393 (3)
C3—C4	1.378 (3)	C13—H13	0.9300
C3—H3	0.9300	C14—C15	1.370 (3)
C4—C5	1.370 (3)	C14—H14	0.9300
C4—H4	0.9300	C15—H15	0.9300
C5—C6	1.373 (3)		
			
C8—O1—N1	105.82 (17)	C2—C7—H7	119.9
C1—N1—O1	103.41 (18)	N2—C8—O1	114.0 (2)
C8—N2—C1	102.7 (2)	N2—C8—C9	129.8 (2)
N4—N3—C10	110.2 (2)	O1—C8—C9	116.2 (2)
N4—N3—C9	120.5 (2)	N3—C9—C8	111.64 (19)
C10—N3—C9	129.1 (2)	N3—C9—H9A	109.3
N5—N4—N3	108.1 (2)	C8—C9—H9A	109.3
N4—N5—C11	108.5 (2)	N3—C9—H9B	109.3
N1—C1—N2	114.0 (2)	C8—C9—H9B	109.3
N1—C1—C2	122.2 (2)	H9A—C9—H9B	108.0
N2—C1—C2	123.8 (2)	N3—C10—C11	104.2 (2)
C3—C2—C7	118.8 (2)	N3—C10—C15	133.4 (2)
C3—C2—C1	120.9 (2)	C11—C10—C15	122.3 (2)
C7—C2—C1	120.2 (2)	N5—C11—C10	108.9 (2)
C4—C3—C2	120.2 (2)	N5—C11—C12	130.5 (2)
C4—C3—H3	119.9	C10—C11—C12	120.5 (2)
C2—C3—H3	119.9	C13—C12—C11	117.0 (2)
C5—C4—C3	120.5 (2)	C13—C12—H12	121.5
C5—C4—H4	119.7	C11—C12—H12	121.5
C3—C4—H4	119.7	C12—C13—C14	121.6 (2)
C4—C5—C6	119.9 (3)	C12—C13—H13	119.2
C4—C5—H5	120.0	C14—C13—H13	119.2
C6—C5—H5	120.0	C15—C14—C13	122.3 (2)
C5—C6—C7	120.2 (3)	C15—C14—H14	118.9
C5—C6—H6	119.9	C13—C14—H14	118.9
C7—C6—H6	119.9	C14—C15—C10	116.2 (2)
C6—C7—C2	120.3 (2)	C14—C15—H15	121.9
C6—C7—H7	119.9	C10—C15—H15	121.9

### Hydrogen-bond geometry (Å, °)

**Table:**

*D*—H···*A*	*D*—H	H···*A*	*D*···*A*	*D*—H···*A*
C9—H9B···N5^i^	0.97	2.59	3.443 (3)	147
C9—H9A···N2^ii^	0.97	2.60	3.466 (3)	149

Symmetry codes: (i) −*x*, *y*+1/2, −*z*+1/2; (ii) *x*−1, *y*, *z*.
